# CXCR4 and CXCR7 Signaling Pathways: A Focus on the Cross-Talk Between Cancer Cells and Tumor Microenvironment

**DOI:** 10.3389/fonc.2021.591386

**Published:** 2021-04-15

**Authors:** Sara Santagata, Caterina Ieranò, Anna Maria Trotta, Anna Capiluongo, Federica Auletta, Giuseppe Guardascione, Stefania Scala

**Affiliations:** Research Department, Microenvironment Molecular Targets, Istituto Nazionale Tumori-IRCCS-Fondazione “G. Pascale”, Napoli, Italy

**Keywords:** CXCR4, CXCR7, tumor microenvironment, immune cells, cancer

## Abstract

The chemokine receptor 4 (CXCR4) and 7 (CXCR7) are G-protein-coupled receptors (GPCRs) activated through their shared ligand CXCL12 in multiple human cancers. They play a key role in the tumor/tumor microenvironment (TME) promoting tumor progression, targeting cell proliferation and migration, while orchestrating the recruitment of immune and stromal cells within the TME. CXCL12 excludes T cells from TME through a concentration gradient that inhibits immunoactive cells access and promotes tumor vascularization. Thus, dual CXCR4/CXCR7 inhibition will target different cancer components. CXCR4/CXCR7 antagonism should prevent the development of metastases by interfering with tumor cell growth, migration and chemotaxis and favoring the frequency of T cells in TME. Herein, we discuss the current understanding on the role of CXCL12/CXCR4/CXCR7 cross-talk in tumor progression and immune cells recruitment providing support for a combined CXCR4/CXCR7 targeting therapy. In addition, we consider emerging approaches that coordinately target both immune checkpoints and CXCL12/CXCR4/CXCR7 axis.

## Introduction

Chemokines are small chemoattractant molecules that control cell migration, proliferation and survival in physiological and pathological processes including cancer (1). They are divided into CC, CXC, XC and CX3C subfamilies based on their cysteine motif (2) and are functionally categorized as inflammatory (CXCL1, CXCL2, CXCL3, CXCL5, CXCL7, CXCL8, CXCL9, CXCL10, CXCL11, and CXCL14) and homeostatic chemokines (CCL14, CCL19, CCL20, CCL21, CCL25, CCL27, CXCL12 and CXCL13) (3). Chemokines act on chemokine receptors, G-protein coupled-7 transmembrane receptors (GPCRs) grouped according to chemokines nomenclature (CCR, CXCR, XCR and CX3CR) (2). About 50 chemokines and 23 human chemokine receptors have been identified (4) including the atypical chemokine receptors (ACKRs), unable to trigger the canonical G protein-signaling and thus called decoys, scavengers or interceptors. Four molecules are included in the ACKR subfamily: ACKR1, or duffy antigen receptor for chemokines (DARC); ACKR2, or D6 or CCBP2; ACKR3, also called CXC-chemokine receptor 7 (CXCR7) or RDC1; and ACKR4, or CC chemokine receptor-like 1 (CCRL1) (5). In TME, both immune cells and stromal cells, express chemokines that regulate tumor vascularization and invasion (6). Herein, the focus is on the CXCL12 that activates CXCR4 and CXCR7. CXCL12, initially known as stromal-derived factor 1 (SDF-1), encoded on chromosome 10q11, is a homeostatic chemokine secreted in lymph nodes, kidney, brain, colon, lung and liver by stromal cells, fibroblasts and epithelial cells in six different isoforms. CXCL12 regulates adhesion of tumor cells with laminin, fibrinogen, stromal cells and endothelial cells (ECs) by activating cell surface adhesion molecules (7, 8). CXCR4 is a co-receptor for Human Immunodeficiency Virus (HIV)-1 entry (9) and binds solely CXCL12, while CXCR7 binds with high affinity CXCL12 and with lower affinity CXCL11 that is also involved in CXCR3 binding (10). CXCL12/CXCR4 axis controls bone marrow (BM) hematopoietic stem cells (HSCs) trafficking (11). CXCL12 transcript and protein levels change periodically in BM with light/dark cycles regulating the retention/mobilization in and from BM of CXCR4-positive HSCs; these cells leave BM during sleep when CXCL12 levels are low and return to BM when CXCL12 increases (12). CXCR7 contributes to the circadian oscillations of CXCL12 within BM and to the neutrophils cycles (13). CXCL12/CXCR4/CXCR7 axis plays a role in cancer regulating cell migration and proliferation, as well as angiogenesis (14). Although molecules targeting CXCR4/CXCR7 have been developed for preclinical and clinical studies in cancer (15), efforts are needed to develop specific and efficient drugs that target both tumor and TME. In this review, we focus on the contribution of the CXCL12/CXCR4/CXCR7 axis in signaling in tumor/TME cells and we evaluate the possible combined targeting of CXCR4 and CXCR7.

## CXCL12/CXCR4/CXCR7 Axis

CXCR4 is a seven-span transmembrane domains (352 amino acids, 48 kDa) GPCRs encoded on chromosome 2.1 (16, 17). CXCR4 is considered a key molecule for normal development as the CXCR4^−/−^ knock-out mice die before birth (18). CXCR4 ^−/−^ knock-out mice show a very low number of mature B and T cells in lymphoid organs and a compromised vascularization in the intestines, stomach, heart and ventricular septal defect that occurs during embryogenesis (19). CXCL12 binding to CXCR4 triggers multiple signal transduction pathways that regulate intracellular calcium flux, chemotaxis, transcription and cell survival (20). CXCL12-CXCR4 forms a complex with the Gαi subunit G protein, inhibiting the adenylyl cyclase–mediated cyclic adenosine monophosphate production and promoting mobilization of intracellular calcium. Gαi subunit dissociates from Gβγ activating Akt, JNK, MEK and ERK1/2 effectors (21). In addition, Gα subunit activates Ras and Rac/Rho pathways, leading to the phosphorylation of ERK and P38 proteins, respectively. CXCR4 homodimerization results in G protein independent activation of the JAK/STAT pathway promoting polarization and chemotactic responses (22). When CXCL12 binds CXCR4, the receptor is modified by ubiquitination before the endocytosis and lysosomal degradation. CXCR4 is desensitized by G proteins uncoupling *via* GPCR kinase (GRK)-dependent phosphorylation and interaction with β –arrestin (23). CXCR7 plays a role in the central nervous system (24), angiogenesis (25), neurogenesis (26) and cardiogenesis (27). Although CXCR7 −/− knock-out mice show a normal hematopoiesis, they die perinatal due to heart malformation, disturbed lymphangiogenesis and cardiomyocyte hyperplasia (28). Initial studies in zebrafish embryos convincingly show a key role of CXCR7 in progenitor cell migration during embryo- and organo-genesis. CXCR7 sequesters CXCL12 from non-target area permitting the correct CXCR4 positive cell migration (29). Without CXCR7, the required CXCL12 gradient for a directional migration is missing thus the migrating cells still respond to CXCL12 but end in undesirable areas (30). CXCR7, as well as CXCR4, is necessary for the correct migration of interneurons and neuronal development and their subcellular location is different: CXCR4 in the plasma membrane and CXCR7 in intracellular recycling endosomes (31). CXCR7 controls CXCL12 signaling in cortical astrocytes and Schwann cells that also express CXCR4. CXCL12-mediated stimulation of astrocytes activates ERK1/2, Akt but not p38, while in Schwann cells CXCL12 activates p38, ERK1/2 and Akt (32). Studies suggest that CXCR7 internalizes CXCL12 and/or CXCL11 inducing intracellular pathways, such as Akt, MAPkinase (MAPK) and JAK/STAT3, through β-arrestin (10) or in heterodimers with CXCR4 (33). CXCR4/CXCR7 complex recruits β-arrestin and activates downstream cell signaling (ERK1/2, p38, SAPK/JNK), inducing cell migration in response to CXCL12 (10). Overall, the CXCR7 signaling relies on cellular context and on relative expression as compared to CXCR4.

## Role of CXCL12-CXCR4/CXCR7 in Cancer

An active CXCL12/CXCR4 pathway is considered a feature of aggressive tumors (34) as it positively correlates with tumor size (17), grading (16), tumor recurrence (35, 36), poor prognosis and patient survival (17, 37, 38). CXCL12/CXCR4 overexpression has been reported in a wide range of tumors such as prostate, brain, breast, lung, liver, colon, ovary and pancreas (39–42). In breast cancer, CXCR4 overexpression promotes tumor cell dissemination to the lungs and lymph nodes (43) while in melanoma, CXCR4 induces lung metastases but not lymph nodes dissemination (44). In non-small cell lung cancer, high CXCR4 expression enhances cellular motility and invasion *via* Epidermal Growth Factor Receptors (EGFRs) and Matrix Metallopeptidase 9 (MMP-9) (45). Also CXCR7 is overexpressed in numerous tumors such as liver, cervical, colon, breast, and pancreatic cancer (46). CXCR7 acts on tumor progression and metastases at different levels upon interaction with endogenous ligands, including CXCL12, CXCL11 and the Macrophage Inhibitory Factor (MIF) (13). The pro-tumorigenic activity of CXCR7 is presumably linked to the interplay with membrane receptors such as estrogen receptor (ER) and EGFR (47, 48). CXCR4 signaling activates mTOR pathway in pancreatic, gastric and renal cancer (49–51). In ovarian cancer, estrogen induces CXCR7 expression that promotes tumor cell migration, invasion and epithelial-mesenchymal transition (EMT) through CXCL11 (52) while CXCL12-stimulated EMT depends on CXCR4, suggesting a context-independent contribution of CXCR7 to EMT-signaling (36). CXCR7 mRNA and protein are overexpressed in colorectal cancer patients and correlate to disease stage and distant metastasis (53). In cervical cancer, high CXCR7 independently correlates to shorter disease-specific survival and it is positively associated with larger tumor size and lymph nodes metastasis (54). In lung adenocarcinoma, CXCR7 expression is considered a poor prognostic marker promoting tumor growth and transforming growth factor-β (TGF-β) mediated EMT (55). Accordingly, CXCR7 together with CXCR4 predicts worse prognosis in renal cell carcinoma patients (56). Conversely, in rhabdomyosarcoma, CXCR7 expression correlates with a less-metastatic phenotype (57). CXCL12 potentiates CXCR7+/CXCR4+ cancer cell trans-endothelial migration toward CCL19 and CXCL13, chemokines expressed by ECs in the lymph nodes (58). In addition, CXCR7 inhibition sensitizes cells to chemotherapy or radiation in murine brain tumors (6). In neuroblastoma, CXCR4 and CXCR7 expression are different or even opposed, as CXCR7 is observed in neural-associated compartment of differentiated and matured tumors while CXCR4 in highly aggressive and undifferentiated tumors. CXCR4 favors neuroblastoma diffusion to liver and lungs, whereas CXCR7 promotes liver and adrenal gland dissemination, both CXCR4 and CXCR7 increase BM invasion (59). In breast cancer, CXCR7 overexpression decreases intravasation thus reducing metastasis while enhancing primary tumor growth *via* angiogenesis (60). Hence, the role of CXCR7 in cancer progression is controversial as some reports suggest pro-metastatic responses and others indicate inhibition of metastasis. The CXCR7-mediated pro-metastatic responses may depend on CXCL11 or on higher receptors availability such as ER (47), EGFR (48) or CXCR4 that significantly contribute to tumor growth and metastasis. CXCR7 regulates CXCR4 surface expression by scavenging CXCL12 (61) or by heterodimerize with CXCR4, reducing CXCR4 internalization and degradation (62), or promoting CXCR4 interactions with intracellular effectors (63). On the other hand, in breast cancer CXCR7 promotes cancer proliferation and angiogenesis but reduces tumor cells intravasation (60). Thus, CXCR7, in the context of high CXCR4, improves chemotaxis to CXCL12 but decreases invasion suppressing CXCL12-induced matrix degradation.

## CXCL12-CXCR4/CXCR7 in the Tumor Microenvironment

**Figure 1:**
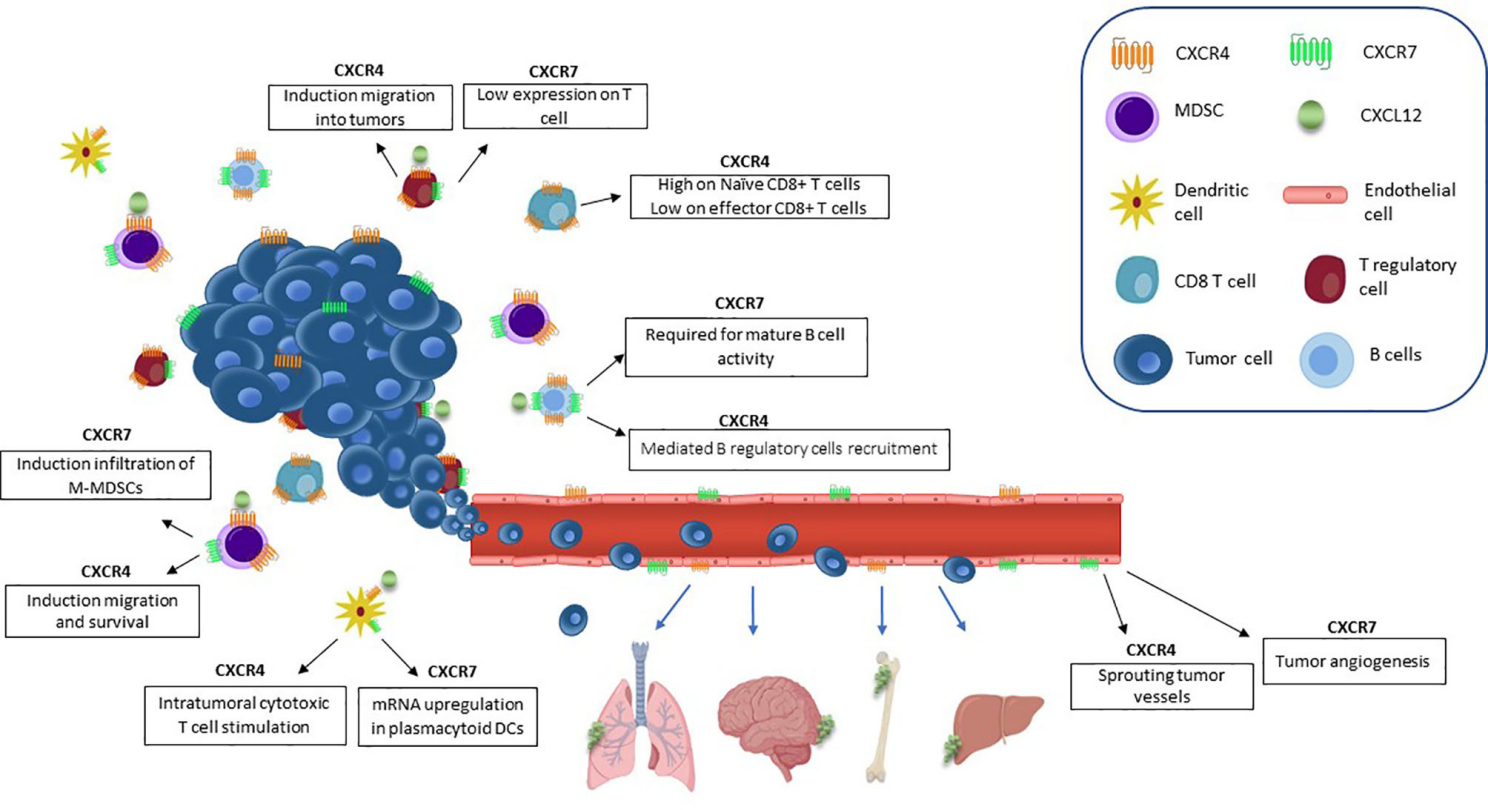
CXCL12/CXCR4/CXCR7 axis in TME. CXCL12 is responsible for TME suppressive cell populations recruitment. CXCL12 induces vascular permeability and allows tumor cell extravasation, thus promoting the metastatic process. ECs CXCR7-positive promote primary tumor growth through secretion of angiogenic factors, such as VEGF. CXCR4 promotes migration and survival of MDSCs and CXCR7 enhances the infiltration of M-MDSCs. The expression of CXCR4 on Tregs promotes intratumoral migration.. CD8+ T cells express CXCR4. Regulatory B cells are recruited to the tumor by CXCL12/CXCR4 and CXCR7 overexpression is involved in the regulation of B cells development and differentiation. Intratumoral CXCR4+ DCs stimulate cytotoxic T cells. Plasmacytoid DCs express CXCR7.

Tumor-derived chemokines are responsible for recruitment of immunosuppressive cells (T regulatory cells (Tregs), myeloid derived suppressor cells (MDSCs), and dendritic cells (DCs) to the tumor niche (64). CXCL12 has an anti-inflammatory role by mediating T cell polarization towards Tregs (65, 66), generating poor functional DCs, and macrophages expressing proangiogenic factors (31). In prostate cancer, high stromal TGF-β induces CXCR4 and activates Akt through stromal CXCL12, thus abrogating the growth-inhibitory responses to TGF-β (67). CXCL12/CXCR4 axis promotes migration and survival of MDSCs in osteosarcoma inhibiting cytotoxic T cell (CTL) expansion and thus controlling tumor growth (68). CXCR7 is highly expressed by tumor associated blood vessels of melanoma, breast and lung cancers, but not by normal vasculature (69). In tumor vascular endothelium, CXCR7 promotes breast, prostate and lung cancer invasive and migratory capability (70). CXCR7 protein is detected in human secondary lymphoid organ-derived B cells, natural killer (NK), basophil and DCs (71, 72). CXCR7 has been reported on CD4+ T cells but not on CD8+ T cells (71). CXCR7 is expressed by lymphocytes and granulocytes in BM and by monocytes, granulocytes, and platelets in peripheral blood. Interestingly, these cells fail to express CXCR7 when isolated from umbilical cord blood (73). In breast cancer, CXCR7 modulates TME-recruiting M2 macrophages through macrophage colony-stimulating factor (M-CSF)/macrophage colony-stimulating factor receptor (MCSF-R) pathway, enhancing tumor growth and metastasis (74). CXCR7/CXCR4 heterodimers promote Monocytic-MDSC (M-MDSCs) and M2-like macrophages in colon cancer turning the TME toward immunosuppression (75). CXCL12/CXCR4/CXCR7 crosstalk in TME is illustrated in **Figure 1**.

## CXCR4 and CXCR7 in Endothelial Cells

In hepatocellular carcinoma (HCC), CXCR4 is expressed in tumor endothelium sprouting tumor vessels (76) and CXCR4-positive ECs predict sorafenib susceptibility. Monocytes/macrophages-TNF-α induces CXCR4 expression on ECs *via* Raf-ERK pathway (77). CXCR7 expression, low in the endothelium, is upregulated during inflammation by pro-inflammatory cytokines such as IL-8 (78) or IL-1b (79), by lipopolysaccharide (80) or during infection by oncoviruses (46). CXCR7 is expressed by ECs and tumor endothelial cells (TECs) promoting their migration (81) and survival (82). It is specifically up-regulated by TNF-α treated/inflamed ECs (83, 84) and is strongly induced by hypoxia-inducible factor-1 alpha (HIF-1α) (85). CXCL12 secreted by TECs, compared to normal endothelial cells (NECs), promotes CXCR7-mediated angiogenesis *via* ERK1/2 suggesting an autocrine/paracrine loop between tumor and TECs (83). Thus, CXCR7 is a promising target for vascular targeted therapies due to its restricted expression and the concomitant effects on leukocytes (e.g., inhibition of immune suppressive Tregs). In contrast, knockout mice with selective depletion of CXCR7 in vascular ECs present more spontaneous lung metastases in “*in vivo*” breast cancer model, indicating that CXCR7 by sequestration of CXCL12 could limit cancer metastases development (86).

## CXCR4 and CXCR7 in Dendritic Cells

DCs are the most potent antigen presenting cells (APCs) in the immune system (87). Immature DCs (iDCs) express CXCR4 to reach inflamed peripheral tissues (88). CXCR4 retains pre-DCs in the BM, CCR2 and CX3CR1 direct migration of pre-DCs to the lung at steady state while CCR2 controls inflammation-directed pre-DCs migration (89). CXCR4 is important for DCs survival, as CXCR4 antagonism reduces mature murine bone marrow-derived DCs (BMDCs) and Langerhans cells (LCs) (90). Plasmacytoid dendritic cells (pDC) secrete type I interferon in response to pathogens while RNA viral natural monoamines/synthetic amines inhibits pDC activation engaging CXCR4 (91). Although CXCR7 mRNA is upregulated in pDCs, it does not correlate with surface protein (71).

## CXCR4 and CXCR7 in T-Regulatory Cells

Tregs (CD4^+^CD25^high^ FoxP3^+^) are CD4+ T cells with predominantly suppressive activity (5–10% of circulating CD4^+^ T cells in humans). Tregs impair immune effector cells function *via* cytokines, direct lysis, inhibitory receptors, metabolic disruption, IL-2 depletion or inducing an immunosuppressive microenvironment (92, 93). Tregs overexpress CXCR4 in advanced cervical cancer (94), malignant pleural mesothelioma (95), ovarian cancer (92) and renal cell carcinoma (96, 97). CXCR4 expression on Tregs correlates with prognosis in ovarian (98), pancreatic (99) and liver cancer (100), or it may not correlate with patient outcome (93, 101). CXCL12 secreted by mesotheliomas attracts CXCR4-positive Foxp3^+^CD25^+^ T cell and is associated with the inflammatory response to these tumors (95). HIF pathway promotes Tregs immunosuppressive function through the expression of their lineage transcriptional regulator FOXP3. In the CXCR4-positive Tregs, tumoral CXCL12 enhances recruitment and suppresses the anti-tumor immune response in basal-like breast cancer (102). CXCR7 is minimally detected but functional on the surface of T cells (69).

## CXCR4 and CXCR7 in CD8 T Cells

CD8+ T cells positively correlate with good prognosis in breast, colorectal, glioblastoma and cervical cancers. In TME, naïve CD8^+^ T cells are differentiated into effector CD8+ T cells and further differentiated into cytotoxic and memory CD8+ T cells (103). CXCR4 is highly expressed in BM on both naive and memory CD8+ T cells where regulates homing to the BM in mice (104). CXCR4 in CD8+ T cells (T^CXCR4^) potentiates migration toward vascular-associated CXCL12-positive cells in the BM. In lymphoma-bearing mice, T^CXCR4^ potentiates the effector function increasing tumor protection (105). In fresh human pancreatic ductal adenocarcinoma (PDAC) slices treated with programmed cell death protein 1 (PD-1) and CXCR4 blockers, CD8+ T cells expansion and apoptosis is detected (106). CD8+ T cells do not express CXCR7 receptor (71).

## CXCR4 and CXCR7 in B Cells

Relatively few B cells are usually found in tumor infiltrates (107). Recent data show that tumor B and plasma cells may exert both pro-tumor and anti-tumor effects depending on the TME, phenotypes of B cells and the relative antibodies production. CXCR4 is expressed at all stages of B cell development in BM from HSCs to mature B cells and plays a major role in the homing of B cell precursors (108). CXCR4 is necessary for developing B cells in the BM but not for mature B cells (109). CXCR4-positive mature B cells home to the BM niche, completing their maturation and staying in contact with CXCL12-expressing BM stromal cells (110). CXCL12/CXCR4 mediates the B regulatory cells recruitment to the tumor inhibiting T cell activity (111). In a spontaneous lymph node metastasis murine breast cancer model, primary tumors induce B cell accumulation in draining lymph nodes. B cells selectively promote lymph node metastasis through pathogenic IgG production that activates the HSPA4-binding protein ITGB5 and the Src/NF-κB pathway in tumor cells for CXCR4/SDF1α-axis-mediated metastasis (112). CXCR7 is expressed in mature B cells and is involved in the regulation of their development and differentiation (69), specifically it is highly restricted to marginal zone B cells and its deletion or CXCR7 specific inhibition, reduces marginal zone B cell numbers and disrupts splenic marginal zone architecture (113). CpG-activated pDCs downregulate CXCR7 expression on primary B cells. CXCR7 expression is required for mature B cells and for the survival and differentiation of the switch memory components, being expressed only in cells that produce antibodies (71). CXCR7 overexpression in Mesenchymal Stem Cells (MSCs) could stimulate regulatory B cells. B cells may negatively regulate tumor immunity and promote tumor progression *via* IL-10 and TGF-β expression (114).

## Targeting the CXCL12/CXCR4/CXCR7 Axis in Combination Therapy

The only approved drug CXCR4 inhibitor is AMD3100 (known as Plerixafor or Mozobil) (115) while multiple antagonists are in different stages of development. CXCR4 antagonists on the field are: (i) modified peptides (BL8040, Balixafortide, FC131); (ii) small-molecules CXCR4 antagonists (AMD3100, AMD11070, MSX-122, GSK812397); (iii) CXCL12 peptide analogs (CTCE-9908, NOX-A12); or (iv) antibodies (MDX-1338/BMS 93656, ALX-0651). BL-8040 promotes infiltration of effector T cells and decreases the number of immune suppressor cells (116). BL-8040 plus the anti PD-1, pembrolizumab, in the COMBAT trial demonstrates that effector T cells potentiate the benefit of chemotherapy in pancreatic ductal adenocarcinoma (PDAC) patients (117). In ovarian cancer, a novel oncolytic vaccinia virus expressing a CXCR4 antagonist (OVV-CXCR4-A-Fc), in combination with DCs pulsed with tumor lysates, can modulate TME by reducing immunosuppressive elements with higher spontaneous antitumor immunity (118). Balixafortide (POL6326) is a cyclic peptide CXCR4 antagonist that effectively mobilizes HSCs. Balixafortide treatment versus eribulin is currently being evaluated in a phase 3 trial after the objective response of balixafortide plus eribulin in the treatment of metastatic Her-negative breast cancers (119). **Table 1** lists CXCR4 inhibitors in clinical development. In contrast to CXCR4 antagonists, there are only few studies describing CXCR7 inhibitors. CCX771 (ChemoCentryx) induces β-arrestin recruitment to the receptor (120), inhibits tumor growth, lung metastasis and tumor angiogenesis *in vivo* (83). In prostate cancer models, CCX771 plus the androgen blocker enzalutamide significantly suppresses tumor growth probably due to low pro-angiogenic signaling (121). Other analogues have been developed with various pharmacological profiles, including the partial agonist CCX777 (122) or CCX733 (ChemoCentryx) which has been reported to act as CXCR7 antagonist (123). The antibody 89Zr-labeled 11G8 is able to detect CXCR7 in mice xenografted with human breast, lung and oesophageal cancers, suggesting that CXCR7 is a viable diagnostic marker (124). Recently, an anti-CXCR7 single chain antibody (X7Ab) with a human immunoglobulin G1 (IgG1) Fc sequence has been described (84). It binds to the same site on the receptor as CXCL12 and inhibits CXCL12-mediated receptor activation. It engages anti-tumor immune response through Fc-driven antibody dependent cell cytotoxicity (ADCC) and antibody-dependent cellular phagocytosis (ADCP) in glioblastoma U343, U251X7, and GL261 cells and, in combination with the temozolomide, significantly reduces glioblastoma progression. Interestingly, commonly prescribed medications atorvastatin and pioglitazone have been shown to decrease CXCR7 expression *via* cholesterol synthesis and peroxisome proliferator-activated receptor (PPAR)-γ respectively, particularly in macrophages (125). Some antagonists could bind both receptors, others bind exclusively CXCR4 or CXCR7 acting as antagonists and/or partial agonist. AMD3100, a CXCR4 antagonist, acts as partial agonist for CXCR7 (126). The cyclic peptidomimetic TC14012 provides therapeutic advantage targeting the CXCR4-CXCL12 axis in chronic lymphocytic leukemia (CLL) while it behaves as CXCR7 agonist in glioma cells (127). A CXCR4 ECL2-based peptide also inhibits CXCR7 internalization (128). Peptide ECL2-X4 displays anti-HIV properties towards CXCR4-using viruses blocking CXCL12 interactions with both CXCR4 and CXCR7.

**Table 1:** CXCR4 antagonists in clinical development.

Drug Name	Phase	Active Indication	Combination Therapy	Trial number
***Small Molecules***				
**Plerixafor (AMD3100)**	Phase 3	Myelokathexis		NCT02231879
	Phase 1	Pancreas, Ovarian, Colon Cancer		NCT3277209
	Phase 2	Metastasis Pancreatic Cancer	Cemiplimab	NCT4177810
	Phase 2	Wiskott-Aldrich Syndrome, Hematopoietic Stem Cell Transplantation		NCT3019809
	Phase 2	Acute Myeloid Leukemia, Acute Lymphoid Leukemia	Busulfan, Cyclophosphamide	NCT2065460
**Mavorixafor (AMD11070; X4P-001)**	Phase 2/3	WHIM Syndrome		NCT03005327
	Phase 1	Waldenestrom’s Macroglobulinemia	Ibrutinib	NCT04274738
	Phase 1	Melanoma	Pembrolizumab	NCT02823405
	Phase 1/2	Renal Cancer	axitinib	NCT02667886
**USL311**	Phase 1/2	Solid Tumors (Phase 1), Relapsed/Recurrent GBM (Phase 2)	Lomustine	NCT02765165
**NOX-A12**	Phase 1/2	Metastatic Colorectal Cancer Metastatic Pancreatic Cancer	Pernbrolizurnab	NCT03168139
**CX-01**	Phase 1	Myelodysplastic Syndromes, Acute Myeloid Leukemia	Azacitidine	NCT02995655
	Phase 2	Acute Myeloid Leukemia	ldarubicin, Cytarabine	NCT02873338
***Peptide CXCR4 antagonists***				
**Balixafortide**	Phase 3	Metastatic Breast Cancer	Eribulin	NCT03786094
**LY2510924**	Phase 1	Leukemia	Idracibin, Cytarabine	NCT02652871
**[68Ga]Pentixafor**	Phase 1	Neuroendocrine Tumors		NCT03335670
	Early Phase	Multiple MyelomaLymphoma		NCT03436342
**BL-8040**	Phase 2	Metastatic Pancreatic Adenocarcinorm	Pernbrolizurnab	NCT02826486
	Phase 2	Malignant Neoplasms of Digestive OrgansMetastatic Pancreatic Cancer	Pernbrolizurnab	NCT02907099
	Phase 3	Multiple Myeloma		NCT03246529
	Phase 1/2	Pancreatic Adenocarcinorm	PEGPH20, Cobimetinib, Atezolizumab, Gemcitabine, Nab- Paclitaxet Oxaliplatin, Leucovorin, Fluorouracil	NCT03193190
	Phase 1/2	Gastric Adenocarcinorm or Gastroesophageal Junction Adenocarcinorm	PEGPH20, Linagliptin, Paclitaxet, Ramucirumab, 5-Fluorouracil (5-FU) Leucovorin, Oxaliplatin, Atezolizurmab, Cobimetinib	NCT03281369
	Phase 1/2	CarcinomaNon-Srmll-Cell Lung AtezolizurmhCobimetinih	Gemicitabine, Carboplatin, Pemetrexed, CPI-444, Tazemetostat, Atezolizumab, Cobimetinib, RO6958688, DOcetaxel	NCT03337698
***Anti-CXCR4 antibodies***				
**Uloccuplumab (MDX-** **1338)**	Phase 1/2	Waldenstrom's Macroglobulinemia	lbtutinib	NCT03225716
	Phase 1/2	Leukemia	Cytarabine	NCT02305563
**ALX-0651**	Phase 1	Healthy Volunteers		NCT01374503
**PF-06747143**	Phase 1	Acute Myeloid Leukemia	Cytarabine, Daunorubicin, Azacitidine, Decitabine	NCT02954653

## Targeting CXCL12/CXCR4/CXCR7 Axis in Combination With Immune Checkpoints Inhibitors (ICIs)

Recently, CXCR4 antagonists have been coupled to ICIs with the intent to remodel TME improving ICIs efficacy (129). Since the initiation of immune checkpoint cascades, such as PD-1 signaling cascade, leads to immune evasion, treatment with ICIs can activate T cells response and enable the immune cells to target tumor cells (130). The lack of immune effector cells, the presence of immune suppressive cells and the polarization of immune cells in the TME play a fundamental role in shifting the balance from an immune active ‘hot’ or ‘T-cell-inflamed TME’ to ‘cold’ TME or ‘non-T-cell-inflamed TME’, such as those from the prostate and pancreas (131, 132). ‘Hot tumors’ are identified by infiltration of T cells and molecular immune activation (133, 134). Chemokines and chemokine receptors represent valuable targets for optimizing antitumor immune responses. In the leukemic hematopoietic microenvironment (LHME) in MLL-AF9-induced mouse acute myeloid leukemia (AML) model, CCL3-CCR1/CCR5 and CXCL12-CXCR4 inhibition block leukemia progression by impairing Tregs migration (135). Combinatorial blockade of CXCR4 and PD-1 reduces Tregs and MDSCs recruitment within the immunosuppressive TME promoting tumor-specific cell-mediated immune responses in ovarian cancer (136). Moreover, CXCR4 blocking inhibits PD-1 expression on CD8+ T cells and promotes the conversion of Tregs into CD4^+^CD25^–^Foxp3^+^IL2^+^CD40L^+^ helper-like cells (137). Inhibition of CXCR4 with AMD3100 decreases desmoplasia, reduces immunosuppression, and improves T cell infiltration and response to ICIs in breast cancer (138) while targeting PD-1 and CXCR4 potentiates anti PD-1 efficacy in murine immune sensitive and immune resistant tumors (139). A nanocomplex of CXCR4 antagonist-paclitaxel-loaded has been developed for pulmonary delivery of anti– programmed death-ligand 1 (PD*-*L1) small interfering RNA (siPD-L1). The nanocomplex promotes T cell infiltration, decreases alpha*-*smooth muscle actin (α-SMA) and collagen, reduces MDSCs and Tregs recruitment (140). Thus, considering CXCR4 and CXCR7 crosstalk in immune cells within the tumor microenvironment, some mechanisms underlying tumor resistance to immunotherapy may be impaired targeting the CXCR4/CXCR7–CXCL12 axis.

## Discussion and Conclusions

Immuno-resistance and vascularization are acquired tumor features that contribute to cancer growth and metastasis. Among the different signaling pathways, directly or indirectly involved in cancer immune-resistance and angiogenesis, CXCR4/CXCR7/CXCL12 is crucial for participating in cancer migration, angiogenesis and immunosuppressive cell recruitment. Thus, the inhibition of the CXCR4/CXCL12 or CXCR7/CXCL12 axis is attractive in cancers overexpressing both receptors such as colorectal cancer (15), renal cancer (51) or glioblastoma (30). Since several CXCR4 antagonists, including peptides, small molecules and antibodies, have been developed and considered for clinical development, the identification of agents able to efficiently block the CXCL12/CXCR7 pathway is still ongoing. However, the observation that CXCR4 inhibition could only partially block the responsiveness of tumor/TME cells to CXCL12 gradients, has questioned the effective role of the exclusive CXCR4/CXCL12 or CXCR7/CXCL12 interaction during cancer progression. CXCL12 inhibitors, such as NOX-012 (141), neutralizing CXCL12 nanobodies (142), or chalcone 4 derivate LIT-927 (143), may affect both CXCR4 and CXCR7 signaling. Unfortunately, the blockage of CXCL12 cannot interfere with CXCR7 signaling mediated by ligands different from CXCL12, such as CXCL11, or cannot provide CXCR4/CXCR7 co-expression, crosstalk and heterodimerization. Thus, despite possible opposite effects should be considered during the design of combination therapies, the administration of antagonists of CXCR4/CXCR7 could offer a valid therapeutic option as a stand-alone therapy or in combination with current immunotherapies.

## Author Contributions

SSa, CI, and SSc contributed in conception and design of the study. AMT, AC, FA, and GG supervised the study. SSa, CI, and SSc wrote and edited the manuscript. SSa and CI equally contributed. All authors contributed to the article and approved the submitted version.

## Conflict of Interest

The authors declare that the research was conducted in the absence of any commercial or financial relationships that could be construed as a potential conflict of interest.
